# 

**Published:** 1903-09-15

**Authors:** 


### CONTENTS.

Our Dental Law, *By Henry K. Lathrop, Jr., D.D.S.* 433

Some Historical and Chemical Facts Relative to the Welding of Gold, *By Henry W. Morgan, M.D., D.D.S.* 441

Our Professional Future, *By C. A. Van Duzee, D.D.S.* 449

Preventive Dentistry, - *By C. M. Wright, D.D.S.* 456

Lower Partial Plates, - - *By John Baur, D.D.S.* 460

Echafolta—its Uses in Dental Surgery, *By B. F. Thielen, D.D.S., Paris, Texas.* 462

Preliminary Announcement of the Fourth International Dental Congress - - - - - - - - 465

Pay no License Fees - - - - - - - 468

Editorial, - - - - - - - - - 470

Indents, - - - - - - - - - 476

